# Goat flock abortion: a retrospective study at Abergelle Agricultural Research Center, Tigray, Ethiopia

**DOI:** 10.1186/s12917-024-03986-0

**Published:** 2024-04-02

**Authors:** Guash Abay Assefa, Teshale Teklue, Mebrahtom Hagazi, Gebretnsae Mezgebe, Weldegebrial G. Aregawi, Adehanom Baraki Tesfaye

**Affiliations:** 1 Abergelle Agricultural Research Center, Tigray Agricultural Research Institute, Abi Adi, Tigray Ethiopia; 2 Mekelle Agricultural Research Center, Tigray Agricultural Research Institute, Mekelle, Tigray Ethiopia; 3 Faculty of Veterinary Medicine, Department of Physiology, Infectiology and Public Health, Ghent University, Ghent, Belgium

**Keywords:** Abortion proportion, Breeding, Goat farm, Tanqua Abergelle district

## Abstract

**Background:**

Small ruminants are the principal component of livestock production in Tigray region, Ethiopia. But their productivity is affected by various factors. According to farmers and expert observation, goat abortion is among the leading causes of production losses in Tanqua-Abergelle district. However, study findings that examine the extent of distribution and economic impact of abortion cases in goats in the district are scarce. This retrospective study investigated the occurrence of abortion and its associated risk factors in three goat breed types at Abergelle Agricultural Research Center goat breeding site over a seven year period. The study included a total of does above one year old, and data were collected from a casebook that was specifically prepared for abortion cases. A thorough follow up was conducted to identify abortion cases. Additionally, a community survey was conducted in selected villages where the research center is located.

**Results:**

The overall abortion proportion was 29.8% in the goat farm. Begait goat breeds had the highest abortion proportion (50.9%, CI 0.36–0.64) in 2015/16. Multivariate logistic regression analysis identified year, season, age and breed as major risk factors of abortion occurrence at flock level. Accordingly, the likelihood of goats experiencing abortion during the dry season (proportion = 34) was 1.87 times higher compared to those in the wet season (proportion = 22.8). Begait breeds had a higher incidence of abortion (proportion 37.5%, OR 4.87, CI 2.49–10.35) compared to other breeds. Age was negatively associated with abortion, suggesting that older goats (OR = 0.67) had a higher relative risk than younger goats (OR = 0.57). Moreover, the study noted a high incidence of abortion during the years 2014/15 to 2016/17 (proportion = 35.7–39.7). Within-breed analysis revealed that age and season were significant risk factors for Abergelle and Begait breeds, respectively by using a multivariate logistic regression analysis. A community survey indicated that 89.7% households responded their goats experienced abortion.

**Conclusions:**

This study highlighted the high prevalence of goat abortion at Abergelle and identifies important risk factors associated with its occurrence. The findings can inform targeted interventions to reduce abortion rates and improve goat productivity in the district.

**Supplementary Information:**

The online version contains supplementary material available at 10.1186/s12917-024-03986-0.

## Background

Small ruminants, sheep and goats, play a vital role in the livelihood of smallholder farmers in Tigray, Ethiopia. The rolling hills of Tigray, carpeted with vibrant bushes and shrubs, offer the perfect grazing ground for sheep and goats to thrive. They are the primary source of meat, milk, manure and savings contributing significantly to the food security [1, 2]. Additionally, small ruminants are the main source of raw skin for the local leather industry (Sheba Leather Industry PLC) that processes and exports skin products of small ruminants in the region, and provide employment opportunity in the region. The local leather industry exports finished leather and leather products such as leather clothes, footwear (shoes, boots and sandals), bags and other leather products from Tigray. According to a Central Statistics Authority of Ethiopia survey [3]; Tigray region possesses 2,097,619 sheep, and 4,838,969 goats. Goats are mainly reared in the lowlands of the Tigray region, such as in Tanqua Abergelle district. Tanqua Abergelle district is known for its prime goat production and is a central hub for goat rearing, with an estimated population of 154,330. Despite their abundance, goat productivity and production in the district fall short of their potential, posing challenges for both the local economy and the wider Tigray region [1, 4].

One of the major constraints to goat production in Tanqua Aberegelle district is high occurrence of abortion cases [5]. Farmers and experts have consistently reported a significant number of abortions annually, raising concerns about the underlying factors contributing to this issue. Only few studies have highlighted the occurrence of abortion in Tigray [6, 7] and in the district [5] respectively. Abortion, defined as the termination of pregnancy before fetal viability [8], can result from various causes, including infectious agents, environmental factors, and management practices. Previous studies in Tigray have identified brucella species as a potential cause of abortion in small ruminants [7], while other research suggests that infectious diseases, extreme weather conditions, feed shortage, physical traumas, and plant poisoning are common causes of abortion in small ruminants of Ethiopia [9].

Infectious agents, such as *Chlamydia psittaci* and *Coxiella burnetii*, have been implicated as major causes of abortion in goats, along with mineral deficiencies, fetal anomalies, and leukoencephalomalacia [10]. Bovine viral diarrhea virus has also been identified as a potential cause, particularly in goats housed with persistently infected cattle [11]. These findings highlight the multi-factorial nature of goat abortion, with infectious agents, environmental factors, and management practices all playing a role. Abortion in small ruminants not only leads to reproductive losses [12, 13] but also poses zoonotic risks, as some causative agents can transmit to humans [14–17].

The Abergelle Agricultural Research Center, established by the Tigray Agricultural Research Institute, is primarily known for its goat breeding and conservation programs. Since 2011, Abergelle, Begait, and their crossbreeds (Abergelle x Begait) have been monitored for performance evaluation. During this period, the flocks were also monitored for susceptibility to different diseases including abortion. Records of abortions within the goat flocks were collected from various evaluation groups. In this report, we present estimates of abortion proportions and their associated risk factors within and among the breeds during the study periods.

## Methods

### Study area description

**Fig. 1:**
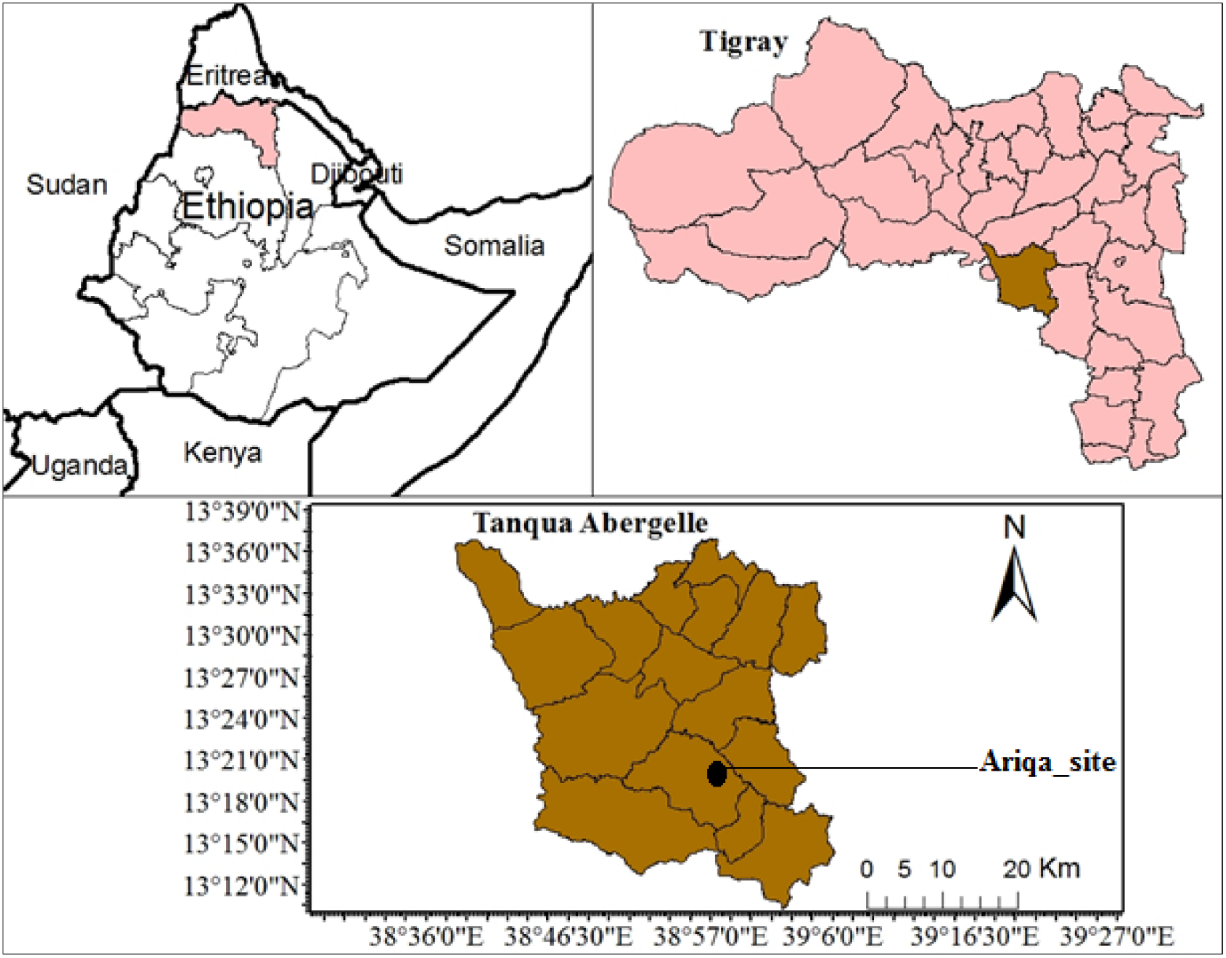
Administrative map of Tanqua Abergelle district, Tigray region, Ethiopia

This study was conducted in Abergelle Agricultural Research Center goat conservation and breeding site called Ariqa site and near community located in Tanqua Abergelle district of Tigray region, Ethiopia. The district is bordered on the south by Amhara region, by Tekeze River from the west, on the north by Kola Tembien, on the east by Degua-Tembien, and on the southeast by the Southeastern zone of Tigray region (Fig. 1). The total area of Tanqua Abergelle district is 1445.63 square kilometers. It extends 13˚ 13.371̍ north latitude and E38˚ 58.856̍ east longitudes. The study districts are categorized as hot to warm sub-moist lowlands of sub-agro ecological zone with an altitude of 1300–1600 m above sea level, and the mean annual rainfall ranges from 400 to 600 mm, which is characterized by low, erratic and variable rainfall. The annual temperature ranges from 28 to 42 ^0^C. The estimated numbers of livestock population in the woreda are to be 69,285 cattle, 154,330 goat, and 83,042 sheep [4]. The dominant goat breed kept is known to be “Abergelle” and are small in size and low milk potential [18]. The production management system is extensive, which is mixed crop farming system. The “Begait” goats are known for their high-quality meat and milk and have bigger size and are originally to be found in the western part of Tigray region [19, 20].

The study animals were three types of goat breeds, namely Abergelle, Begiat and their crosses. All does above and equal to one year old were included in this study regardless of their breeds. These does assigned to breeding activity based on their breeding status, body condition and health status. The sample sizes differed every year based on the number of does give birth, maturity, and culling of the goats. To all breeds, a semi-intensive management system is practiced at the center with the flock grazing and browsing outdoors during the day (7:30 − 5:30 AM and 2:00–5:30 PM) and housed in pens (with half concrete building walls with mesh wire and corrugated metal sheet roofs) at night. Hay and water are freely available to the goats but additional supplementary feed (inclusive of wheat bran, “nug” cake (type of pulse), molasses, lime and salt) also provided depending on the age and physiological status of the animal.

Cross-breeding program is an alternative genetic improvement tool, applied using two different breeds of high (Begait) and low (Abergelle) genetic potentials. Bucks are selected based on their general health status and presence of abnormalities. Accordingly, Bucks with good general health and no visible abnormalities (such as small testes or hocked joints) are selected and then randomly assigned to doe groups. Mating groups are assigned twice a year (June and December) and 15 to 20 does were assigned to a single buck and usually lasts for about 42 days (minimum of two oestrous cycles) and controlled single sire mating is practiced.

### Data collection

A seven-year abortion data set, recorded from September 2006 to August 2012 Ethiopian Calendar (2013/14-2019/2020 Gregorian Calendar), was used for the analysis. The Ethiopian year starts in September and ends in August. Every case of abortion was recorded in a separate casebook each year by an employed enumerator. For each animal that aborted, data on age, parity, season, date of abortion, breed, and stage of abortion were promptly recorded. Every year, this data was transferred from the case book to a computer and then used for this study.

Seasons were categorized as dry and wet based on local weather observations. Age was categorized as young (≤ 2 years old) and adult (> 2 years old). The study included all does that were at least one year old and exposed to breeding every year. Bucks were assigned to breeding groups of does based on the breeding activity’s objective. In this case, the breeding farm aims to conserve pure Abergelle breeds and improve their milk and meat production capacity by crossbreeding them with Begait goats. There were three breed categories at the research center: pure Abergelle, pure Begait, and their crosses (Abergelle x Begait). Parity was categorized as nullparous/primiparous (≤ 1 parity) and multiparous (> 1 parity) according to Ullah et al. [21].

A community-level random field survey was conducted through interviews to assess the situation of goat abortion in Tanqua Abergelle districts in 2017/18. A total of 29 farmers participated in the interview.

### Data analysis

A descriptive statistical analysis was used to assess the state of goat abortion in the community and the abortion proportion on the farm. Abortion proportion was calculated by dividing the number of positive animals (numerator) by the total number of animals at risk (denominator) at the end of the study year according to [9]. Goats included in the denominator were those in the above-one-year age range assigned for breeding.

Factors such as breed, parity, age, season, and study year were considered independent variables, while abortion proportion was the dependent variable.

The association between risk factors and the dependent variable (abortion proportion) was analyzed using the chi-square test in R software. The level of significance was set at 5% and the confidence level at 95%.

The second part of the statistical analysis employed univariable and multivariable logistic regression analysis in R software. The univariable analysis was conducted for breed-related and animal-level variables, where each variable was tested one by one using the “glm” function from the “lme4” package. Variables that differed significantly in the univariable analysis (*P* < 0.05) were included in the multivariable analysis. Multicollinearity and confounding factors were also checked by determining the variance inflation factor using the “vif” function from the “car” package. An individual VIF above 10 and/or an average VIF above 1 was considered indicative of significant multicollinearity. Based on this analysis, our independent variables were independent of each other. Model fitness was assessed using the Chi-square goodness of fit and deviance residuals. The backward stepwise approach is a statistical modeling method that involves the iterative removal of the least informative explanatory variable from an initial model containing all possible variables. The process continues until a final and optimal model is achieved [22].

## Results

### Descriptive analysis of the overall abortion proportion and their associated risk factors

The abortion figures of goats for a seven-year period, from September 2013 to August 2020, are presented in Table 1. The estimated overall percentage of abortion during the study period was 29.8% (95% confidence interval [CI] 0.27–0.33). Descriptively, the overall percentage of abortion was highest for the Begait breed (37.5%; CI 0.32–0.43), followed by the Abergelle breed (27.3%; CI 0.24–0.31) and its crosses (13.2%; CI 0.06–0.23). The abortion proportions for all breeds were higher in the years 2014/15 to 2017/18 than in the other years. Figuratively, the overall abortion proportions during the study period were high. Seasonal changes showed a significant impact on the occurrence of abortions for each breed where abortion proportion during the dry season was estimated to be 34% as compared to the wet season (22.8%). Additionally, the percentage of abortion in goats less than or equal to two years of age and goats older than two years was estimated at 36.4% and 27.8%, respectively. A chi-square analysis identified a significant associations between abortion occurrence and the following factors: study year, season, breed (*P* ≤ 0.001), and age (*P* ≤ 0.05). Parity, however, was not significantly associated (*P* > 0.05).

Concerning to the stage of abortion, 52.7% (154/292) of goats experienced early abortion, while 47.3% (138/292) experienced late abortion.

**Table 1:** Abortion proportions across the study years and estimated risk factors

		Breed (‘***’)							
Variables	Category	Abergelle		Begait		Cross		Total	
		Pos./n	Proportion. %(95%CI)	Pos./n	Proportion. %(95%CI	Pos./n	Proportion. %(95%CI)	Pos./n	Proportion. %(95%CI)
**Parity**									‘_’
	Null/primi	54/155	34.8 (0.27–0.43)	27/65	41.5 (0.29–0.54)	7/48	14.6 (0.06–0.28)	88/273	32.2 (0.26–0.38)
	Multiparous	99/405	24.4 (0.20–0.29)	102/279	36.5 (0.31–0.43)	3/28	10.7 (0.02–0.28)	204/707	28.8 (0.25–0.32)
**Year**									‘***’
	2013/14	11/71	15.5 (0.08–0.26)	6/45	13.3 (0.05–0.26)	2/2	100(0.16-1)	19/118	16.1 (0.01–0.24)
	2014/15	26/82	31.7 (0.22–0.43)	19/42	45.2 (0.29–0.61)	5/14	35.7 (0.13–0.65)	49/137	35.7 (0.27–0.44)
	2015/16	30/78	38.5 (0.27–0.50)	20/43	46.5 (0.31–0.62)	0/5	0 (0.00-0.52)	50/126	39.7 (0.31–0.49)
	2016/17	32/85	37.6 (0.27–0.49)	26/52	50 (0.36–0.64)	0/8	0 (0.00-0.37)	58/146	39.7 (0.32–0.48)
	2017/18	25/88	28.4 (0.19–0.39)	21/54	38.9 (0.26–0.53)	3/18	16.7 (0.04–0.41)	49/160	30.6 (0.24–0.38)
	2018/19	20/90	22.2 (0.14–0.32)	23/57	40.4 (0.27–0.54	0/16	0 (0.00-0.21)	43/163	26.4 (0.19–0.34)
	2019/20	11/67	16.4 (0.08–0.27)	14/51	27.5 (0.16–0.42)	0/13	0 (0.00-0.25)	24/130	18.5 (0.12–0.26)
**Season**									‘***’
	Wet	47/213	22.1 (0.17–0.28)	35/132	26.5 (0.19–0.35)	2/23	8.7 (0.01–0.28)	84/368	22.8 (0.18–0.27)
	Dry	106/547	19.4 (0.16–0.23)	94/212	44.3 (0.37–0.51)	8/53	15.1 (0.07–0.27)	208/612	34 (0.30–0.38)
**Age**									‘*’
	≤ 2 year	52/143	36.4 (0.28–0.45)	26/61	42.6 (0.30–0.56)	5/24	20.8 (0.07–0.42)	83/228	36.4 (0.30–0.43)
	> 2 year	101/417	24.2 (0.20–0.28)	103/283	36.4 (0.31–0.42)	5/52	9.6 (0.03–0.21)	209/752	27.8 (0.25–0.31)
**Total**		**153/560**	**27.3 (0.24–0.31)**	**129/344**	**37.5 (0.32–0.43)**	**10/76**	**13.2 (0.06–0.23)**	**292/980**	**29.8 (0.27–0.33)**

Significance codes: ‘***’ 0.001, ‘**’ 0.01, ‘*’ 0.05, ‘_’ non-significant

Where; n = sample size, Pos. = number of positive animals, Proportion. %= proportion in percent

The wide probability intervals provide poor support for the risk factors evaluated. Some of the predictors for breeds (considering crosses) had small sample sizes where further data and analysis may allow for better estimation in the future.

**Fig. 2:**
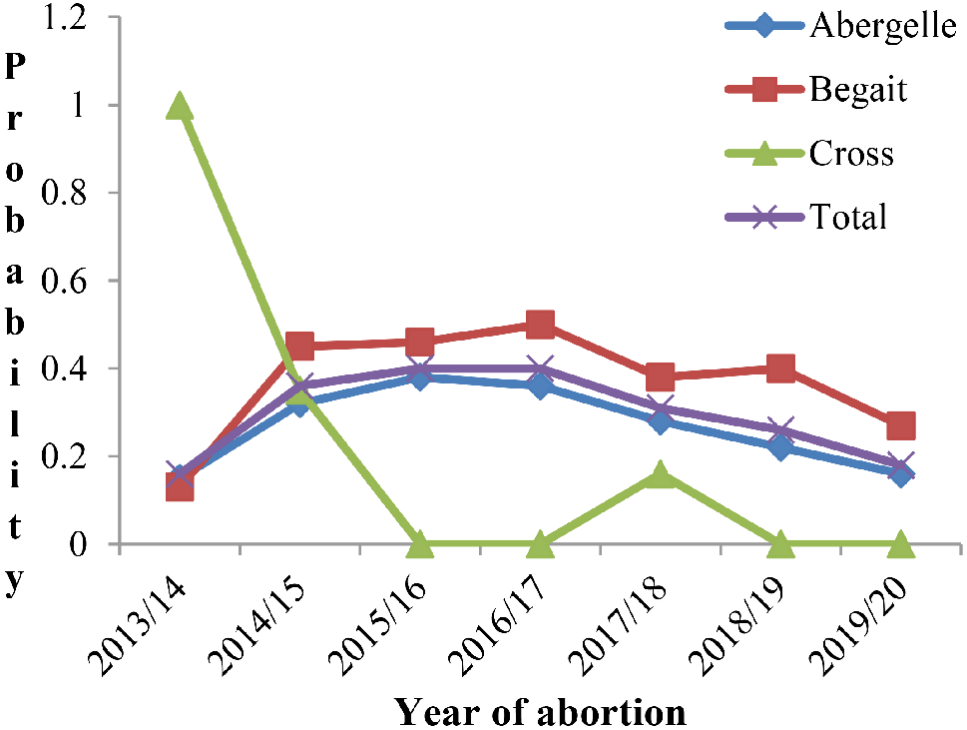
The predicted probabilities of abortion proportion for the three breeds across years

The predicted probabilities of the abortion proportions for the three breeds in the different study years were also estimated (Fig. 2). The predicted probabilities of abortion between breeds aligned in direction and magnitude across the study years except for the cross breeds. As indicated in the Fig. 2, the proportions of abortion increased until 2016/17 and only started to dropdown at the end of 2019/20 compared to the previous years, similar to what is explained in the table above.

### The abortion proportion between breeds and its associated risk factors

In the second part of statistical analysis (Table 2), using generalized linear model approach tested each variable one by one in each model, variables year (*P* ≤ 0.0001), season (*P* ≤ 0.0001), age (*P* < 0.05), and breed (*P* ≤ 0.001) were significantly associated with abortion occurrence. Conversely, parity was not significantly associated (*P* > 0.05).

Univariable logistic regression analysis revealed that goats in the dry season (OR 1.74, CI: 1.28–2.37, *P* ≤ 0.0001) had a higher likelihood of experiencing abortion compared to those in the wet season (OR 0.29, CI 0.23–0.37). The likelihood of experiencing abortion increased each year when compared to the reference year (2013/14 G.C), with the highest proportions observed from 2014/15 to 2016/17, slight decrease from the years 2017/18 to 2019/20. Regarding breeds, Begait breed (OR 3.5, CI 1.85–7.23) followed by the Abergelle breed (OR 2.22, CI 1.2–4.55) exhibited higher abortion proportions compared to the cross breeds (reference group) (OR 0.17, CI 0.08–0.31). Additionally, goats that were two years old or younger had lower odds for experiencing abortion (OR = 0.57) than goats older than two years (OR = 0.67). Both coefficients being negative (-0.56 for ≤ 2 year and − 0.0.40 for > 2 year), indicating that increasing age is associated with a decreased risk of abortion.

**Table 2:** Univariable regression analysis of the abortion proportion across associated risk factors

Variable	Category	ß (estimate) (coefficients)	Std. error	Odds ratio (e^ß^)	95% CI (e^ß^)	P-value
Parity						
	Null/primi	-0.74	0.13	0.47	0.37–0.61	
	Multiparous	-0.16	0.15	0.85	0.63–1.15	0.3
Year						
	2013/14	-1.65	0.25	0.19	0.11–0.31	
	2014/15	1.06	0.307	2.9	1.6–5.4	0.000
	2015/16	1.23	0.309	3.43	1.89–6.41	0.000
	2016/17	1.23	0.302	3.43	1.93–6.34	0.001
	2017/18	0.83	0.303	2.3	1.28–4.25	0.01
	2018/19	0.62	0.307	1.86	1.03–3.47	0.05
	2019/20	0.16	0.337	1.18	0.61–2.31	0.6
Season						
	Wet	-1.22	0.124	0.29	0.23–0.37	
	Dry	0.55	0.15	1.74	1.28–2.37	0.000
Age						
	≤ 2 year	-0.5579	0.1376	0.57	0.44–0.75	
	> 2 year	-0.3969	0.1599	0.67	0.49–0.92	0.013
Breed						
	Cross	-1.77	0.32	0.17	0.08–0.31	
	Abergelle	0.79	0.33	2.22	1.2–4.55	0.02
	Begait	1.25	0.34	3.5	1.85–7.23	0.000

A backward stepwise approach was used to assess model fitness based on the pearson chi-square test and the deviance table. Initially, the model included all available explanatory variables. However, parity was not statistically significant. Therefore, parity was removed from the model, which was then re-fitted. The resulting chi-square test was highly significant (*P* ≤ 0.0001), indicating that we should reject the null hypothesis that the standard model (without parity) is the correct model.

The multivariate logistic regression analysis presented in Table 3 identified year, season, age of abortion, and breed type as the relevant risk factors affecting the occurrence of abortion during the study periods. Compared to the univariate regression analysis, removing the variable parity resulted in a stronger significance level, odds proportion, and coefficients. As a result, the major findings are as follows: During the dry season, goats are1.87 times more likely to abort compared to the wet season. The odds of Begait and Abergelle breeds having an abortion are 4.87 and 2.68 higher, respectively, compared to the crossbreed. Concerning the age, goats aged over 2 years has approximately 43% reduced odds abortion compared those under the ages of less than or equal to 2 years. Concerning the year of abortion, goats in 2019/20, 2018/19, 2017/18, 2016/17, 2015/16, and 2014/15 were 1.64, 2.62, 3.15, 4.6, 4.14, and 3.78 times, respectively, were more likely to abort than those in 2013/14.

**Table 3:** Multivariable logistic regression analysis of the abortion proportion between breeds across associated risk factors

Variable	Category	ß (estimate) (coefficients)	Std. error	Odds ratio (e^ß^)	95% CI (e^ß^)	P-value
Year						
	2013/14					
	2014/15	1.33	0.32	3.78	2.05–7.22	0.000
	2015/16	1.42	0.32	4.14	2.24–7.91	0.000
	2016/17	1.53	0.32	4.6	2.52–8.73	0.000
	2017/18	1.45	0.32	3.15	1.71-6.00	0.000
	2018/19	0.96	0.32	2.62	1.42-5.00	0.003
	2019/20	0.49	0.35	1.64	0.82–3.30	0.16
Season						
	Wet					
	Dry	0.63	0.16	1.87	1.38–2.57	0.000
Age						
	≤ 2 year					
	> 2 year	-0.64	0.15	0.53	0.37–0.75	0.000
Breed						
	Cross					
	Abergelle	0.98	0.35	2.68	1.40–5.62	0.004
	Begait	1.58	0.36	4.87	2.49–10.35	0.000

### Within breed evaluation of abortion proportion and its associated risk factors

The chi-square analysis revealed that parity and season differed significantly (*P* < 0.05) whereas age and years were highly significantly associated (*P* ≤ 0.01) with the occurrence of abortion at animal level in Abergelle goat breeds. In Begait goat breeds, parity and age were not significantly associated (*P* > 0.05) whereas season (*P* ≤ 0.001) and year (*P* ≤ 0.01) were significantly differed to abortion proportion. For the Cross goat breeds; none of the estimated risk factors were significantly associated with the proportion of abortions (*P* > 0.05).

The univariable logistic regression analysis presented in Table 4 shows that the abortion proportion for Abergelle breeds, increased from 2013/14 to 2016/17 but gradually declined from 2017/18 to 2019/20. Goats were significantly more likely to abort during the dry season (OR 1.55, CI 1.023–2.46) compared to the wet season (OR 0.28, CI 0.20–0.38). A backward stepwise approach to assess model fitness identified age as the only statistically significant factor (*P* < 0.01) and it was retained in the alternative model. Interestingly, age is still negatively associated with the occurrence of abortion where both age groups had reduced odds of abortion by approximately 43%.

**Table 4:** Univariable logistic regression analysis of the abortion proportion and their associated risk factors for Abergelle goat breeds

Variables	Category	ß (estimate) (coefficients)	Std. error	Odds ratio (e^ß^)	95% CI (e^ß^)	P-value
Parity						
	Null/primi	-0.63	0.16	0.53	0.38–0.74	
	Multiparous	-0.5	0.2	0.61	0.41–0.91	0.013
Year						
	2013/14	-1.6	0.33	0.18	0.09–0.33	
	2014/15	0.89	0.41	2.43	1.12–5.58	0.03
	2015/16	1.22	0.4	3.41	1.58–7.76	0.002
	2016/17	1.17	0.39	3.23	1.52–7.29	0.003
	2017/18	0.77	0.4	2.16	1.00-4.94	0.06
	2018/19	0.44	0.41	1.56	0.70–3.61	0.3
	2019/20	-0.02	0.47	0.97	0.38–2.48	0.9
Season						
	Wet	-1.26	0.16	0.28	0.20–0.38	
	Dry	0.44	0.2	1.55	1.05–2.32	0.03
Age						
	≤ 2 year	-0.56	0.17	0.57	0.40–0.79	
	> 2 year	-0.58	0.21	0.56	0.37–0.84	0.005

Similar to Abergelle goats, the proportion of abortion in Begait goat breeds (Table 5), increased over the years with the exception for a decrease in 2019/20. Additionally, goats were significantly more likely to abort during the dry season (OR 2.2, CI 1.38–3.57) compared to the wet season (OR 0.36, CI 0.24–0.52). A backward stepwise approach identified season as the only significant factor (*P* ≤ 0.001) for inclusion in the alternative model. Therefore, the multivariable regression analysis identified season as the most significant risk factor among those analyzed. Goats were 2.2 times more likely to abort during the dry season compared to the wet season.

For the cross breeds, none of the risk factors was significantly different.

**Table 5:** Univariable logistic regression analysis of the abortion proportion and their associated risk factors for Begait goat breeds

Variable	Category	ß (estimate) (coefficients)	Std. error	Odds ratio (e^ß^)	95% CI (e^ß^)	P-value
Parity						
	Null/primi	-0.34	0.25	0.71	0.43–1.16	
	Multiparous	-0.21	0.28	0.81	0.47–1.42	0.45
Year						
	2013/14	-1.87	0.43	0.15	0.06–0.34	
	2014/15	1.68	0.53	5.37	1.96–16.55	0.001
	2015/16	1.73	0.53	5.65	2.07–17.36	0.001
	2016/17	1.87	0.51	6.49	2.47–19.46	0.000
	2017/18	1.42	0.51	4.13	1.56–12.37	0.006
	2018/19	1.48	0.51	4.39	1.68–13.05	0.004
	2019/20	0.89	0.53	2.46	0.88–7.57	0.09
Season						
	Wet	-1.02	0.19	0.36	0.24–0.52	
	Dry	0.79	0.24	2.2	1.38–3.57	0.001
Age						
	≤ 2 year	-0.29	0.25	0.74	0.44–1.23	
	> 2 year	-0.26	0.28	0.77	0.44–1.36	0.36

### Participatory interview

To support our findings from the breeding site, we conducted unstructured questionnaire survey at the community level (outside the research farm) (Table 6**).** Nearly 90% (26 out of 29) of the interviewed households reported goat abortions.

Importantly, 26 farmers reported multiple abortions: two or more goats aborted per household. Notably, one household reported a significant loss of 20 goat kids due to abortion. Heat stress and drought were reported as primary cause of abortion by most farmers with only seven mentioning physical trauma. The hot season emerged as the highest risk period, mentioned by 25 times respondents. Notably, the first trimester (44.8%) was identified as the most vulnerable pregnancy stage for abortion, followed by the third trimester (34.50%). Regarding the consequence on does, difficult birth and decrease milk production were reported most frequently (each 31.0%), followed by placental retention (17.20%). Interestingly, 65.50% of respondents believed goats experiencing their first pregnancy (first kidding) were at higher risk of abortion. Awareness of potential zoonotic risks from contact with aborted animals was remarkably low (86.2%), with only four household expressing concerns. Additionally, our corresponding author (Guash Abay) observations, along with reports from farmers and experts, suggest that a single abortion event, especially during the dry season, can affect multiple pregnant does within the same flock.

**Table 6:** Questionnaire survey results to the status of abortion in the study districts

Parameters	Category	Frequency	Percent
Abortion experience in their flock			
	Yes	26	89.7
	No	3	10.3
Frequency per animal			
	Once	24	82.8
	More than 2	5	17.2
Season of occurrence			
	Hot	25	86.2
	Rainfall	4	13.8
Pregnancy stage at risk			
	First trimester	13	44.8
	Second trimester	6	20.7
	Third	10	34.5
Dam effect			
	Difficult birth	9	31
	No return to birth	2	6.9
	Milk decrease	9	31
	Dam illness	4	13.7
	Retained placenta	5	17.2
Age			
	Doeling	19	65.5
	Adults	10	34.5
General loss due to abortion			
	Loss of kid	10	33.3
	Loss of dam	5	16.7
	Loss of milk	5	16.7
	all	7	23.3
Zoonosis awareness			
	No	25	86.2
	Yes	4	13.8

Key: doeling = for goats under or equal to the age of 2 year, adult = for goats above the age of 2 years

## Discussion

**Fig. 3:**
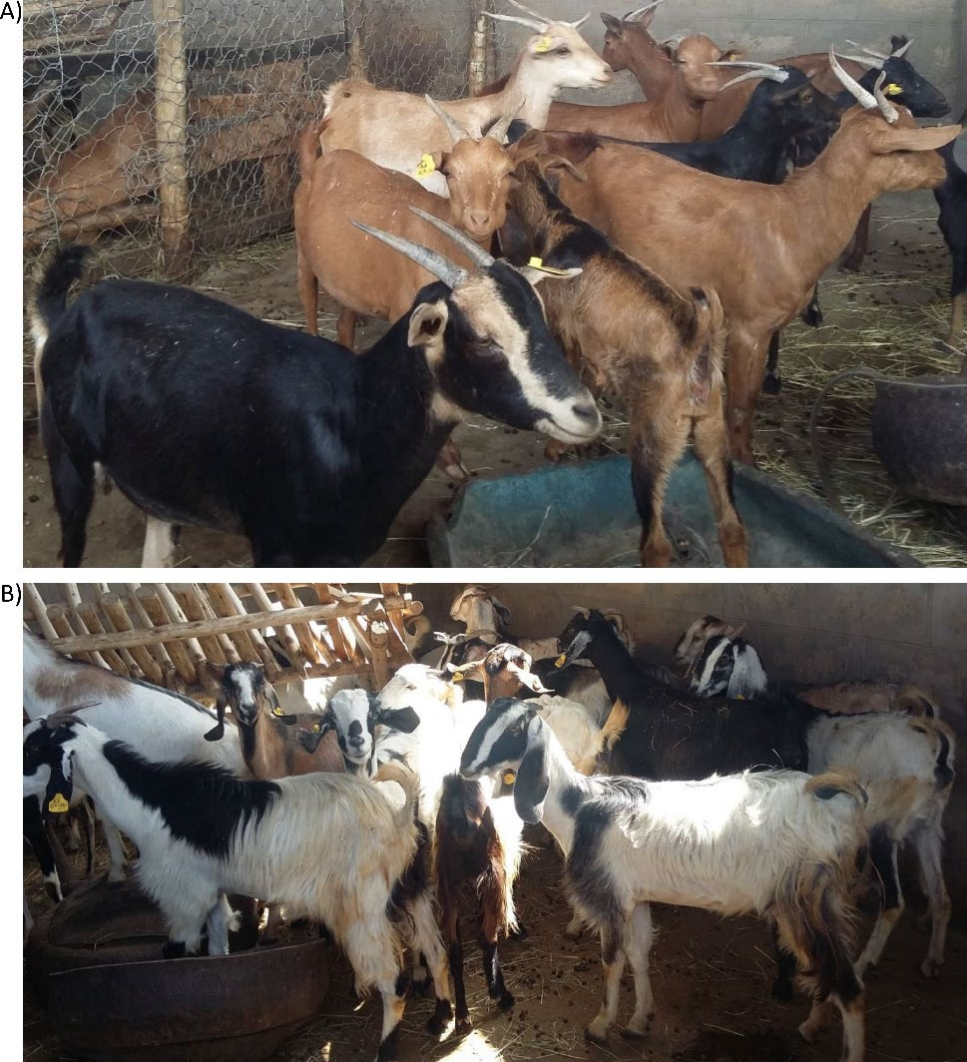
Some figurative description of the type of breeds in the farm: **A**) Abergelle goat breed type **B**) Begait breed goat type

Animal health interventions are key to support the breed improvement through different reproductive technologies, such as cross breeding and selection in our case [23]. The Abergelle Agricultural Research Center goat breeding site evaluates the genetic performance of the three breeds (Pure Abergelle, Pure Begait, and their Crosses (Abergelle x Begait) (Fig. 3)) to optimize the meat demand of the society as well as the country at large. Goat reproductive problems such as abortion were among the prioritized issues evaluated and monitored during the study periods.

A high proportion of abortion (29.8%) was observed in a goat breeding farm with a semi-intensive management system. Furthermore, a preliminary community survey revealed an even higher proportion of abortion (overall 89.7%) among goat flocks in the area employing an extensive (traditional) management system. These findings align with reports from other parts of Ethiopia: with [24] reported 57.5%, and [9] reported 58.68%. However, Alamerew et al. [25] observed a lower proportion of 19.5% in North Shoa, Central Ethiopia. Globally abortion storms have been reported in various countries as well. For example, in Morocco [26], 10.26% of does were reported to be affected, while in Algeria [27], the proportion reached 75.33% for both sheep and goats, and 34% caprine abortion in the Netherlands [16]. Notably, Abubakar et al. [28] identified a high proportion of abortions in goats infected with Peste des Petitis Ruminant (PPR). According to Menzies [29], abortion proportions exceeding 5%, especially when concentrated in a short time period or specific location, warrant immediate investigation.

A sustained 2–5% range might indicate endemic causes. Considering this, the high prevalence suggests the potential abortion factors within the farm and the surrounding community, demanding further investigation.

Our discussion with district veterinarians and farmers, focusing on abortion in small ruminants, confirmed the high frequency of occurrence and a strong desire for investigation from the farmers. Notably, farmers reported simultaneous abortions affecting multiple pregnant does within flocks, particularly during the dry season when feed and rainfall are limited. This led many to believe that these abortions are caused by “devil attacks,” a local belief that devils can harm people and animals during hot weather and nights.

Unfortunately, due to limited laboratory capacity both within the region and at our research institute, we were unable to investigate potential infectious or mineral deficiency causes further. Veterinary laboratories are centralized in Addis Ababa, the capital city of Ethiopia, which is 1000 km away from the study area. Furthermore, limited budgets, a shortage of veterinary personnel, and insufficient equipment and reagents for sample collection and transportation (e.g., transport mediums) in both organizations significantly hindered our efforts to transport samples to Addis Ababa for laboratory analysis.

Seasonal differences significantly impacted the occurrence of abortion in the goat farm. The district experiences a long dry season (Sep-May) with inconsistent rainfall during June to August leading to feed shortages and repeated droughts (Guash Abay, personal observation). Additionally, very high rates of abortions (abortion storms) were observed annually during specific months: September, and March to mid-June. September marks the beginning of the dry season, while March to mid-June are the hottest months of the year with scarce feed resources (Guash Abay, personal observation). Overall, proportion of abortions remained high during the study period, despite some variation between years. There was a slight reduction of abortion during the later three years (2017/18-2019/20) though it wasn’t a significant reduction. This could be attributed to gradual improvement in managemental practice such as feeding, husbandry and bio-security to pregnant goats, as well as culling of goats with poor health or productivity. Our findings aligned with [29] who reported increased goat abortions during the dry period. Additionally, several studies have linked climate changes, including rising temperatures and erratic rainfall patterns, to abortion outbreaks in goats, including in Ethiopia [9] and South Africa [30].

Breed difference significantly affected proportion of abortion occurrence. Begait breeds originally from different agro-ecology in western part of Tigray that were undergoing adaptation trial, exhibited a notably higher abortion proportion (37.5%) compared to other breeds. This suggests begait breeds might be more susceptible to abortion in the study environment. Similar breed-related differences in abortion susceptibility have been reported in goat farms elsewhere, including Namibia [31] and Mexico [32].

For Abergelle breeds, only age significant influenced abortion risk, while for Begait breeds, season was the key factor. Notably, previous studies on Abergelle studies focused on their niche environment, while Begait studies took place outside their usual habitat. These differences along with confounding factors, could explain the contrasting results observed in our study.

Despite the potential role of bacterial and viral zoonotic agents in abortion, a staggering 86.2% of the community lacked awareness about zoonotic diseases linked to animal abortion. This significant knowledge gap stems from limited public education and awareness programs in the district on zoonotic diseases. Researches in Ethiopia have confirmed the significant impact of zoonotic pathogens on small ruminant abortions. For instance, studied by [25] identified Q-fever and Toxoplasmosis as prevalent causes, while others like [33] and [34] found Chlamydia spp., *Coxiella burnetii*, and *Toxoplasma gondii* as infectious causes. Notably [7], even detected Brucella species in small ruminants in southern Tigray. Similar trends are observed globally. In the Netherlands, Q fever has been linked to goat farm outbreaks [16, 35], while Caprine alphaherpesvirus 1 emerged as a major cause in Spain [36].

In the study district (community level), the risk of transmission of zoonotic infectious agents is high due to traditional farming systems. Goats and sheep are reared together in the same flocks, without separate management systems such as isolating sick animals or providing specific feeding and housing for pregnant does and kids. Additionally, the proximity of animal and human housing facilities increases the potential for disease transmission. Moreover, newly purchased animals are introduced to the flock without implementing preventive measures such as quarantine and vaccination.

## Conclusions

In conclusion, our study identified alarmingly high proportion of abortions among goats both on our farm and in the surrounding community. This explains to significant economic losses through kid mortality and reduced milk production. Breed and season were identified as the most likely factors influencing abortion occurrence within the farm, with Begait goats showing particular susceptibility. Additionally, limited awareness of zoonotic causes of abortion was observed among community members. While our study was limited by its retrospective nature and the absence of laboratory investigations or a representative community survey, it highlights the importance of abortion as a factor hindering small ruminant production and productivity in the region. We strongly recommend further investigation to identify the specific causative agents of abortion, quantify the associated economic losses, and inform the development of targeted intervention strategies.

### Electronic supplementary material

Below is the link to the electronic supplementary material.

Supplementary Material 1

Supplementary Material 2

Supplementary Material 3

Supplementary Material 4

## Data Availability

All data are available upon request from the authors.
