# Supplementary figures and images for: Goat flock abortion: a retrospective study at Abergelle Agricultural Research Center, Tigray, Ethiopia
Source: BMC Vet Res. 2024 Apr 2;20:132. doi: 10.1186/s12917-024-03986-0 (PMC10985995; doi:10.1186/s12917-024-03986-0)

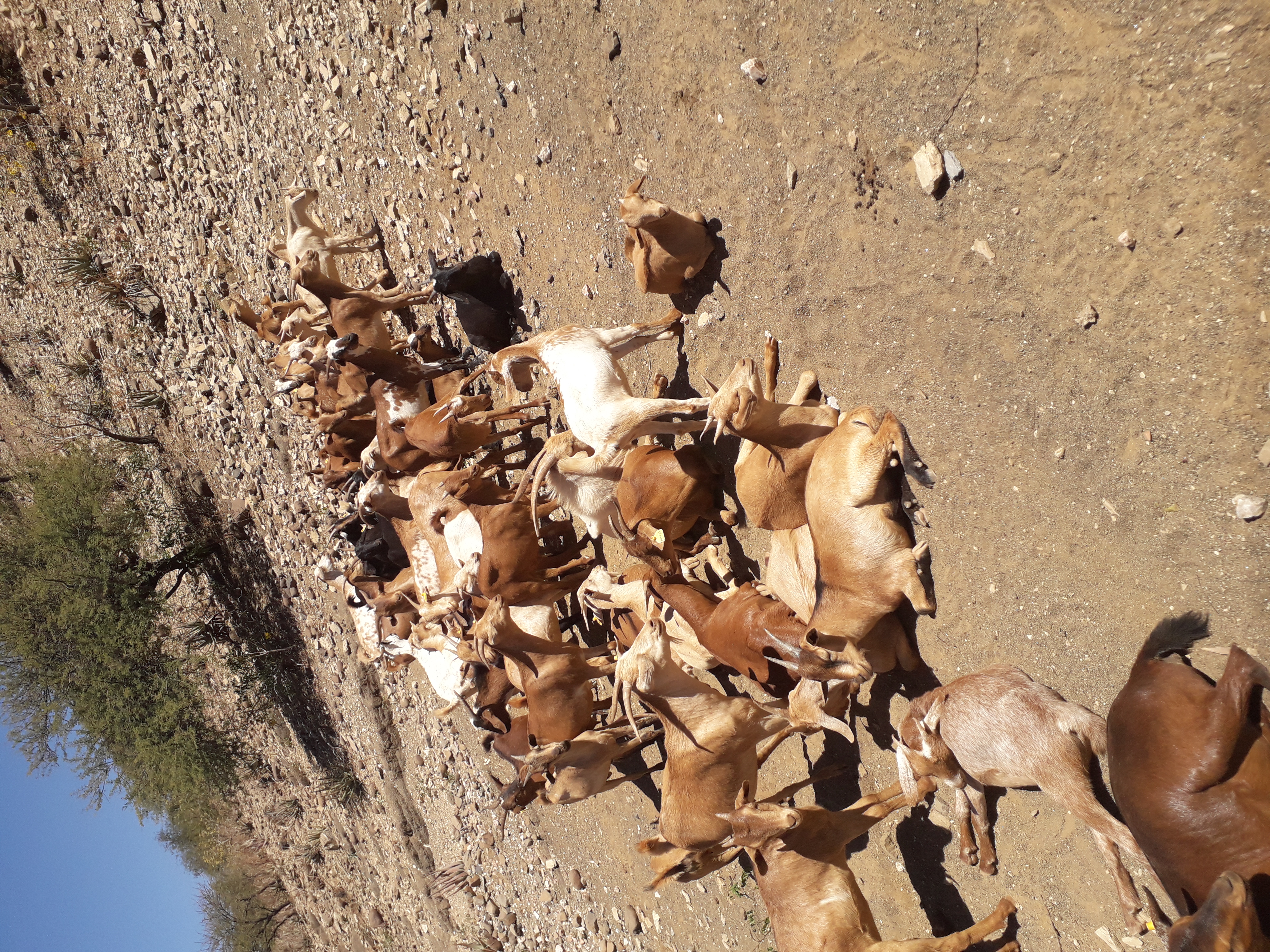

Supplement: Supplementary file 1 — Supplementary Material 1 [file 12917_2024_3986_MOESM1_ESM.jpg]

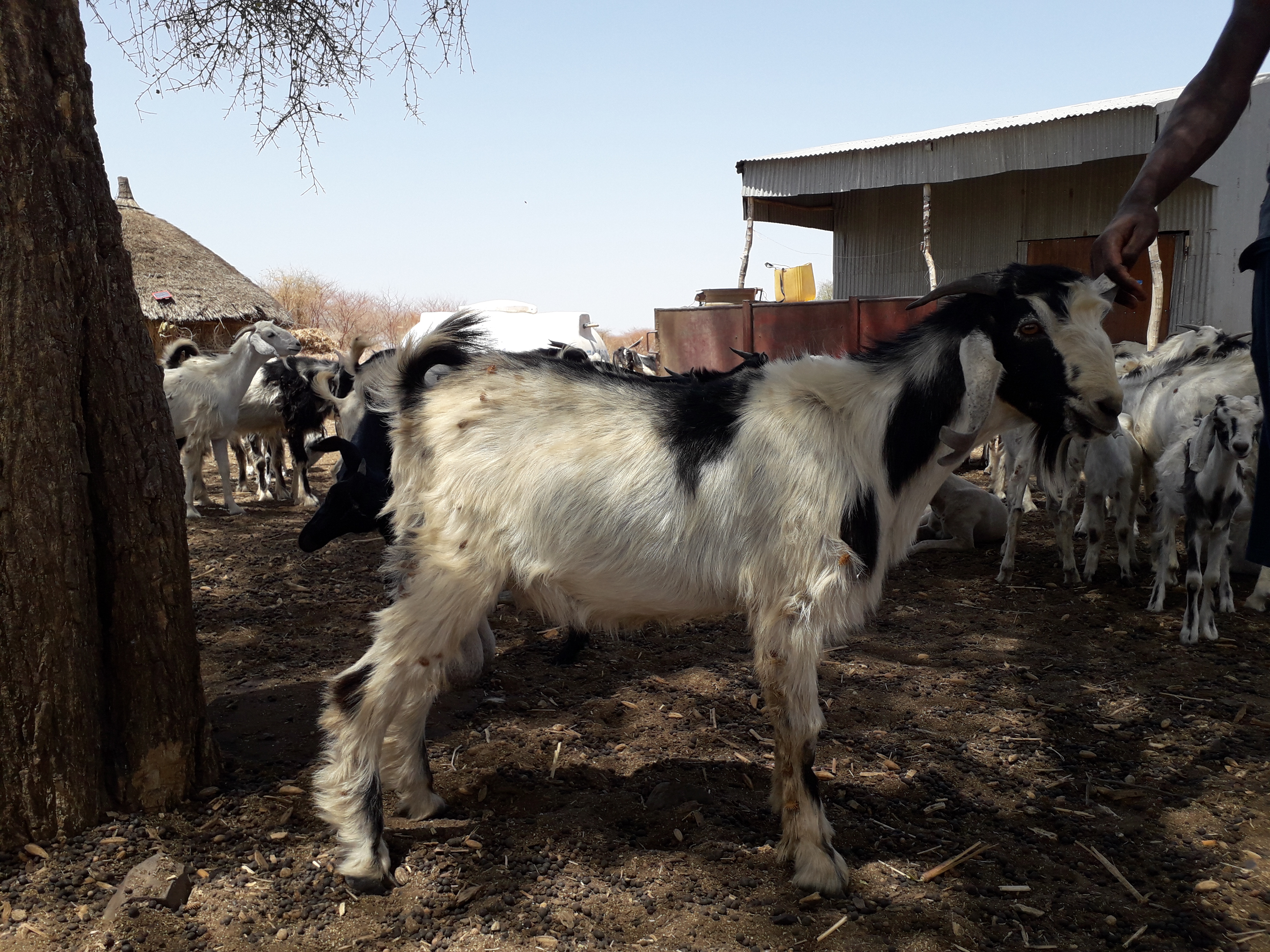

Supplement: Supplementary file 2 — Supplementary Material 2 [file 12917_2024_3986_MOESM2_ESM.jpg]

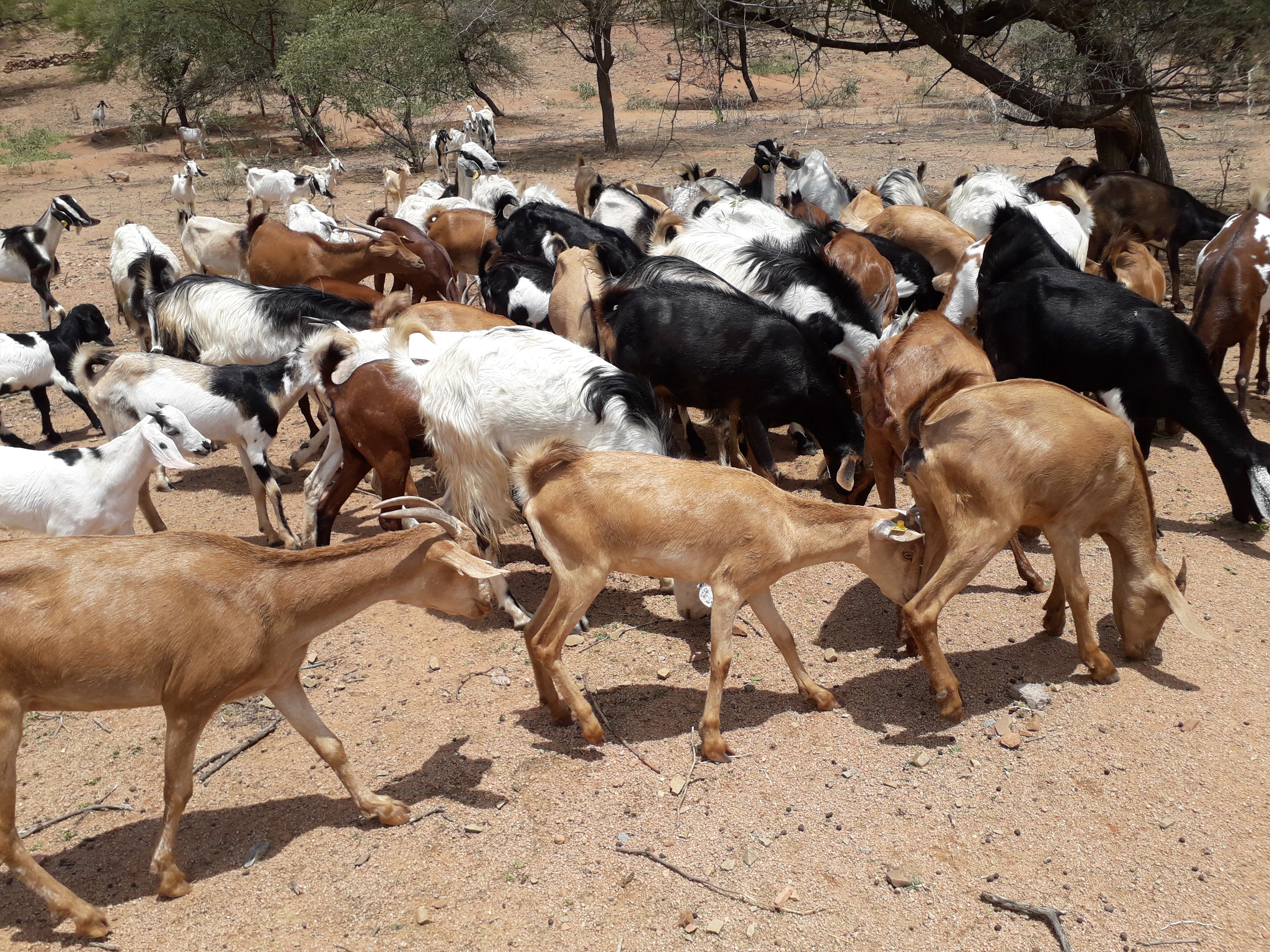

Supplement: Supplementary file 3 — Supplementary Material 3 [file 12917_2024_3986_MOESM3_ESM.jpg]
