# Supplementary material for: Goat flock abortion: a retrospective study at Abergelle Agricultural Research Center, Tigray, Ethiopia
Source: BMC Vet Res. 2024 Apr 2;20:132. doi: 10.1186/s12917-024-03986-0 (PMC10985995; doi:10.1186/s12917-024-03986-0)
Supplement: Supplementary file 4 — Supplementary Material 4 [file 12917_2024_3986_MOESM4_ESM.doc]

Abortion cases record sheet at Abergelle Agricultural Research Center goat conservation and breeding site (Ariqa site)

| Doe ID | Age | Parity | Date of abortion | Stage of abortion | Sex | Reasons of abortion | Remarks |
| --- | --- | --- | --- | --- | --- | --- | --- |
|  |  |  |  | 1. early  2. late  3. still | 1.M  2. F | 1. Injury  2. Other |  |
|  |  |  |  |  |  |  |  |
|  |  |  |  |  |  |  |  |
|  |  |  |  |  |  |  |  |
|  |  |  |  |  |  |  |  |
|  |  |  |  |  |  |  |  |
|  |  |  |  |  |  |  |  |
|  |  |  |  |  |  |  |  |
|  |  |  |  |  |  |  |  |
|  |  |  |  |  |  |  |  |
|  |  |  |  |  |  |  |  |
|  |  |  |  |  |  |  |  |
|  |  |  |  |  |  |  |  |
|  |  |  |  |  |  |  |  |
|  |  |  |  |  |  |  |  |
|  |  |  |  |  |  |  |  |
|  |  |  |  |  |  |  |  |
